# Predicting Voltammetry Using Physics-Informed Neural Networks

**DOI:** 10.1021/acs.jpclett.1c04054

**Published:** 2022-01-10

**Authors:** Haotian Chen, Enno Kätelhön, Richard G. Compton

**Affiliations:** † Department of Chemistry, Physical and Theoretical Chemistry Laboratory, Oxford University, South Parks Road, Oxford OX1 3QZ, U.K.; ‡ MHP Management- und IT-Beratung GmbH, Königsallee 49, 71638 Ludwigsburg, Germany

## Abstract

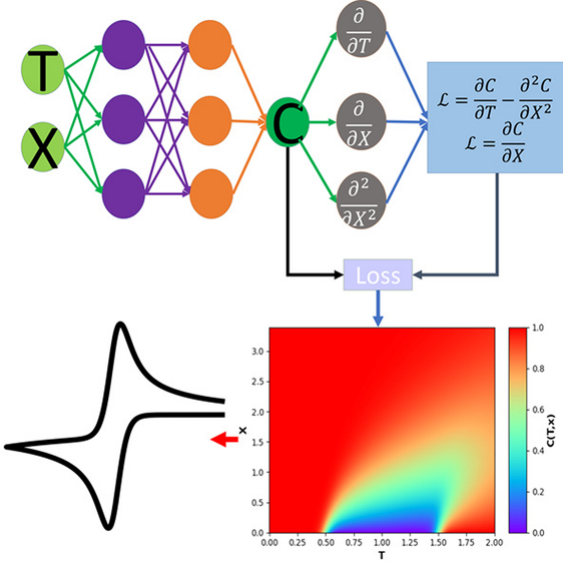

We propose a discretization-free approach to simulation of cyclic voltammetry using Physics-Informed Neural Networks (PINNs) by constraining a feed-forward neutral network with the diffusion equation and electrochemically consistent boundary conditions. Using PINNs, we first predict one-dimensional voltammetry at a disc electrode with semi-infinite or thin layer boundary conditions. The voltammograms agree quantitatively with those obtained independently using the finite difference method and/or previously reported analytical expressions. Further, we predict the voltammetry at a microband electrode, solving the two-dimensional diffusion equation, obtaining results in close agreement with the literature. Last, we apply a PINN to voltammetry at the edges of a square electrode, quantifying the nonuniform current distribution near the corner of electrode. In general, we noticed the relative ease of developing PINNs for the solution of, in particular, the higher dimensional problem, and recommend PINNs as a potentially faster and easier alternative to existing approaches for voltammetric problems.

Physics-informed neural networks (PINNs), introduced by Raissi in 2018 to solve the Burger, Schrodinger, and Alan–Cahn equations among others,^1^ form a class of neural networks that can integrate data and abstract mathematical operators, along with the laws of nature, to provide physically consistent solutions. PINNs have been applied widely in modeling, analysis, and parameter estimation to lithium-ion batteries^2−5^ and fuel cells.^6−8^ A PINN can also be informed by chemical kinetics^9,10^ and thermodynamics^11^ to solve the partial differential equations (PDEs) that describe diverse physical chemical models. With the growing application of PINN in scientific and engineering contexts, we note that there are no reports applying PINNs to solve electrochemical problems with coupled diffusional mass transport both in general and in particular in the context of voltammetry, the most generally applied electrochemical methodology.^12,13^ Simulation of cyclic voltammetry conventionally employs finite difference,^14^ finite element,^15,16^ or random walk algorithms,^14^ all needing discretization of simulation spaces leading to the difficulty that the requirements of discretization/simulation grow exponentially as simulations expand to higher dimensions.^17^ In the following, a discretization-free simulation, empowered by data driven inference of PDEs using PINNs, of 1D and 2D cyclic voltammetry with Nernstian, no-flux, or fixed concentration boundary conditions is developed, offering a radically new approach to the modeling of voltammetry.

Cyclic voltammetry applies a time dependent potential, *E*, as a triangular waveform to a working electrode with measurement of the resulting current as a function of the applied potential resulting in a “voltammogram” in the form of a plot of current vs potential. The proofs-of-concept in this article focus on the simple electrode processes of the form:
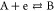
1where A and B are stable, solution phase species. We assume that mass transport in solution to the macro electrode can be described as diffusion in one dimension with Fick’s second law of diffusion:^18−20^
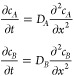
2Here *D*_A_ and *D*_B_ are the diffusion coefficients for A and B respectively. In the present work, the diffusion coefficients of both species are assumed to be equal, so solving only for species A is sufficient for simulation of cyclic voltammetry since it is easily shown that for this condition the sum *c*_A_ + *c*_B_ = *c*_A_^*^ where *c*_A_^*^ is the bulk concentration of A and the bulk concentration of B is assumed to be zero.

We further assume that the boundary condition on the surface of electrode can be described using the Nernst equation:
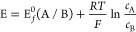
3where E and *E*_*f*_^0^, are, respectively, the applied potential and the formal potential of the A/B couple. *R*, *T*, and *F* are the gas constant, temperature and the Faraday constant, respectively. For cyclic voltammetry, the triangular waveform of potential *E* is expressed as
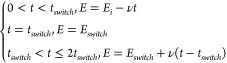
4where *E*_*i*_ is the starting potential of the forward scan, *E*_*switch*_ is the potential at which the triangular sweep reverse direction, *t*_*switch*_ is the start time of the reverse scan, and ν is the potential scan rate (V/s). Positive and negative potential scan rates are usually chosen corresponding to the study of reduction and oxidation processes, respectively.

We first apply PINNs to predict two problems in cyclic voltammetry involving one spatial dimension (*x*). In the first of these, we consider cyclic voltammetry at a macroelectrode, specifically a disc electrode of radius, *r*_*e*_, with semi-infinite diffusion to allow comparison with the well-known response reported in many textbooks.^12,21^ Second, we examine the thin-layer cyclic voltammetry corresponding to one-dimensional diffusion within a fixed and finite volume such as applied within thin layer cells^22,23^ as are commonly encountered in spectro-electrochemistry.^24^ We then, second, consider two problems involving two-spatial dimensions, (*x*, *y*). The first is cyclic voltammetry at a microband electrode where comparisons with previous finite difference simulations can be made. Second, we investigate a novel problem involving the two-dimensional diffusion to the edges of a square electrode, as shown in Figure 1. The continuous time model proposed by Raissi et al. is used.^1^

**Figure 1:**
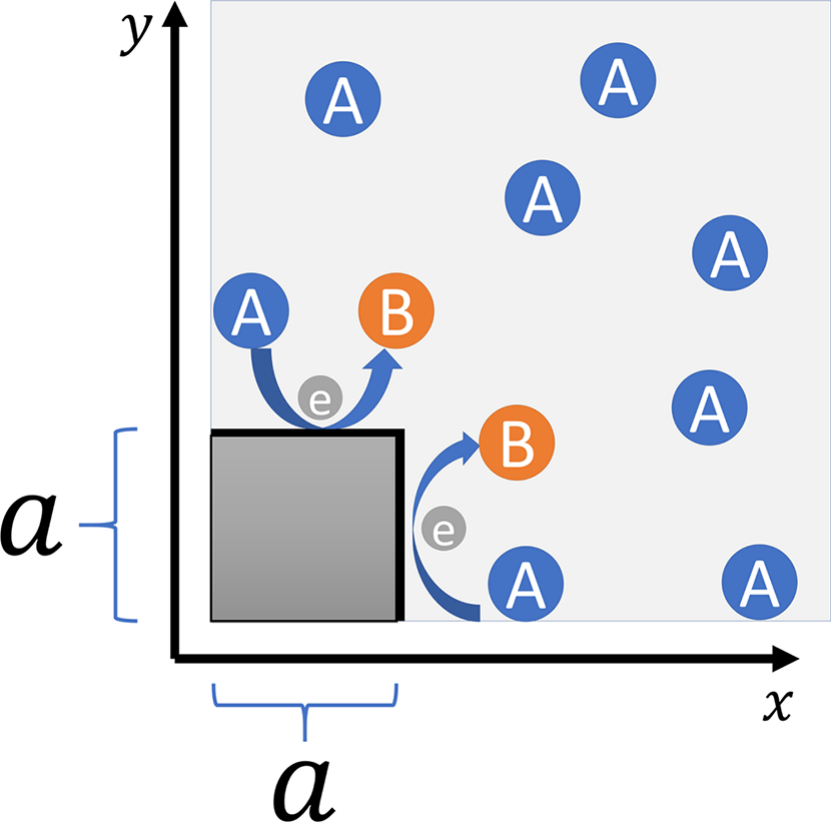
Scheme of cyclic voltammetry at the edges of a square electrode with edge length *a*. Species A and B are free to diffuse in the *x*, *y* plane and to interconvert electrochemically at the two edges of the square electrode. The electrochemical reaction of A to B is reversible.

The modeling parameters for use in the PINNs are first converted to dimensionless form. Dimensionless parameters (see Table 1) are used to remove the dependence on bulk concentration, c*, electrode size (radius, *r*_*e*_, for the disc electrode; the width, *w*, for the microband electrode; and the edge length, *a*, of the square), and diffusion coefficient, *D*, so predictions are general as one prediction of the PINNs is compatible with any set of c*, electrode size, and *D*. Using dimensionless parameters can also reduce the vanishing/exploding gradient problem commonly encountered during training of neural networks.^25^ The range of scan is noted as [*θ*_*i*_, *θ*_*switch*_], and *θ*_*i*_ and *θ*_*switch*_ are the starting potential and reverse potentials, respectively. The time for the full scan is thus  where σ is the dimensionless scan rate. The maximum spatial distance in the coordinate *X* for simulation is guided by Einstein’s work on Brownian motion:^26^ the root-mean-square displacement of a particle is . To ensure that the outer boundary is sufficiently remote as not to be affected by diffusion the outer boundary of the simulation, *x*_sim_ is located at  in dimensional form and equivalent to  in dimensionless form.^27^ At the electrode surface, since the dimensionless concentrations of A and B always sum to 1 at all points in space within the equal diffusion coefficient approximation, the Nernst equation predicts the dimensionless concentration of A as a function of dimensionless potential θ:
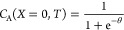
5

**Table 1:** Dimensionless Parameters for the 1D Disc, the 2D Microband, and the 2D square Electrode Simulations^a^

parameter	1-D disc	2-D microband	2-D square edges
concentration	*C*_*j*_ = *c*_*j*_/*c*_A_^*^	*C*_*j*_ = *c*_*j*_/*c*_A_^*^	*C*_*j*_ = *c*_*j*_/*c*_A_^*^
diffusion coefficient	*d*_*j*_ = *D*_*j*_/*D*_A_	*d*_*j*_ = *D*_*j*_/*D*_A_	*d*_*j*_ = *D*_*j*_/*D*_A_
spatial coordinates	X = *x*/*r*_*e*_	X = *x*/*w*, *Y* = *y*/*w*	X = *x*/*a*, *Y* = *y*/*a*
time	*T* = *D*_A_*t*/*r*_*e*_^2^	*T* = *D*_A_*t*/*w*^2^	*T* = *D*_A_*t*/*a*^2^
potential	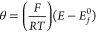	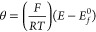	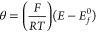
scan rate	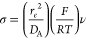	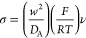	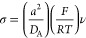
current	*J* = *I* /*πrFc*_A_^*^D_A_	*J* = *I* /*Fc*_A_^*^D_A_*b*	*J* = *I* /Fc_A_^*^D_A_*a* (each edge)

a *c*_*j*_, *D*_*j*_ are respectively the concentration and diffusion coefficients of species *j*. *r*_*e*_, *w*, *b*, and *a* are the radius of the disc electrode, the width and the length of the microband electrode, and the edge length of the square electrode, respectively.

The PINNs modeling of 1D voltammetry requires prediction of the evolution of the dimensionless concentration of A, *C*(*T*,*X*), as a function of dimensionless time *T* and dimensionless distance *X* from the electrode surface and is described by the dimensionless diffusion equation and boundary conditions in the following form:
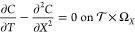
6.1
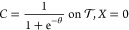
6.2
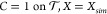
6.3
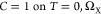
6.4where , Ω_*X*_ ∈[0,*X*_*sim*_ ]represents the temporal and one-dimensional spatial domains, respectively. Since the diffusion coefficients of A and B are assumed equal, the dimensionless diffusion coefficients are always 1 and so do not appear in the diffusion equation. Equations 6.2 and 6.3 are Dirichlet boundary conditions at the surface obeying the Nernst equation and at the outer boundary with a fixed concentration, respectively. Equation 6.4 represents the initial state at *T* = 0. Four training data sets are required to enforce the physical laws in the form of the diffusion equation and boundary conditions. For example, to enforce eq 6.1, a set of *N* collocation points {*T*_*i*_,*X*_*i*_ }_*i*=1_^*N*^ is generated using a random uniform distribution throughout the temporal–spatial domain. Similarly, the data points for the Nernst boundary condition implied by eq 6.2 is also a set of N collocation points {*T*_*i*_,0 }_*i*=1_^*N*^ randomly uniformly distributed only in the temporal domain. The data points for eq 6.3 and 6.4 are {*T*_*i*_,*X*_*sim*_ }_*i*=1_^*N*^ and {0,*X*_*i*_ }_*i*=1_^*N*^ respectively.

Unlike a conventional neural network trained to predict *C*(*T*,*X*) from (*T*,*X*) with an enormous amount of known concentrations in , in a PINN, a dense neural network is used to approximate the unknown solution *C*(*T*,*X*) with only known concentrations at the boundaries (*X* = 0 or *X*_*sim*_) and for the initial state (*T* = 0). More importantly, eq 6.1 has to be satisfied for every point inside the whole  domain. To enforce the physics given by eq 6.1, and the known concentrations at the boundary and initial state, a loss function  is composed as a linear combination of four mean square error (MSE) functions:
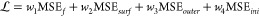
7where *w*_*j*_ are hyperparameters for training to balance the weights of each MSEs since each MSEs may have different numerical scales. The first MSE_*f*_ term represents the error of enforcing the diffusion equation as
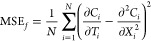
8where *C*_*i*_ is the concentration predicted by the neural network from (*T*_*i*_,*X*_*i*_); and  are the gradients calculated by the machine learning framework. The three other MSEs are the errors of the predicted concentrations at the boundary. For example, MSE_*surf*_ represents the error of predicting the concentrations at the surface of electrode as
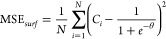
9and MSE_*outer*_ and MSE_*ini*_ are the errors of predicting the concentration at the outer boundary and at the initial state when *T* = 0. During training, the optimizer of the neural network minimizes the combined loss function  by tuning the weights and biases of the neural network. Adam^28^ was used as the optimizer with a learning rate of 10^–3^.

After training, the neural network predicts *C*(*T*,*X*) for cyclic voltammetry. For cyclic voltammetry with a dimensionless scan rate of σ = 40, the concentration is plotted as a function of *T* and *X* in Figure 2a. Figure 2a shows that *C* decreases with time near the surface of electrode (*X* = 0) when 0.25 < *T* < 0.75, which is expected since the overpotential θ is negative between 0.25 < *T* < 0.75 so reduction of A to B at the surface of electrode surfaces lowers the surface concentration. When *T* > < 0.75, A is regenerated via oxidation of B to A, observing a local high concentration of A near the electrode surface. The dimensionless flux,  and d*X* is a small spatial step. Figure 2b compares the voltammogram predicted by PINN with simulation using the finite difference method.^14^ The forward scan peak flux is also compared with the Randles–Ševčík equation^29,30^ in dimensionless form as , the excellent agreement with both comparisons showing that the PINN, provided with only the information on the boundary conditions and the diffusion equation, can accurately predict the concentration profile and thus the voltammogram.

**Figure 2:**
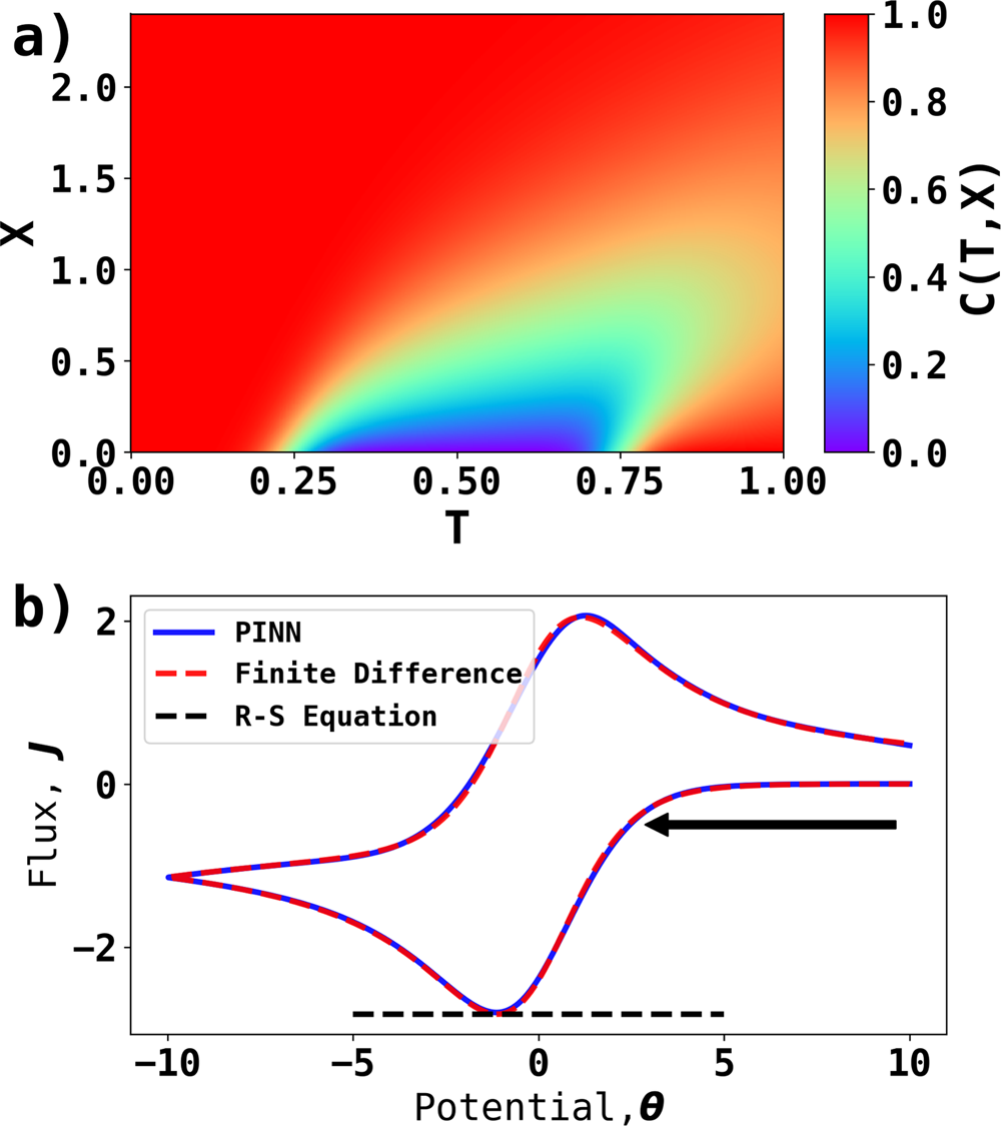
Prediction results of PINN for cyclic voltammetry at σ = 40. (a) Temporal–spatial concentration profile and (b) the voltammogram predicted by PINN (blue line) compared with the voltammogram generated using finite difference method (red dashed line). The peak current is compared with the peak current predicted using the Randles–Ševčík equation (black dashed line). The black arrow indicates the start the initial direction of scan.

A PINN can also be applied to solve cyclic voltammetry with a Neumann boundary condition which we illustrate for thin-layer cyclic voltammetry. Thin-layer cyclic voltammetry is different from normal cyclic voltammetry by having a constrained diffusion layer. In experimental practice the constraint is provided by solid wall opposite the electrode through which material cannot pass.^31,32^ Mathematically this corresponds to a no-flux boundary condition at the outer boundary of simulation. The diffusion equation and boundary conditions can be expressed as
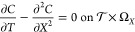
10.1
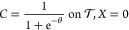
10.2
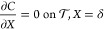
10.3
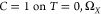
10.4where the thin layer cell (dimensionless) thickness, δ, also the outer boundary of simulation, defines the scale of the cell thickness to the extent of the diffusion layer via  where λ is a factor signaling thin-layer behavior (small λ) and semi-infinite behavior (large λ). The spatial domain Ω_*X*_ is defined as  for PINN prediction. The no-flux boundary condition can be enforced by
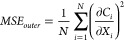
11where *X*_*i*_ = δ represents the outer boundary for the PINN prediction.

Using PINN, the electrochemically reversible cyclic voltammetry when σ = 40 was simulated corresponding to a thin layer factor λ = 0.035. The temporal–spatial concentration profile predicted by the PINN is shown in Figure 3a, and the corresponding cyclic voltammogram is shown in Figure 3b. Figure 2a shows the thin-layer concentration pattern with A almost fully converted to B when 0.25 < *T* < 0.75.

**Figure 3:**
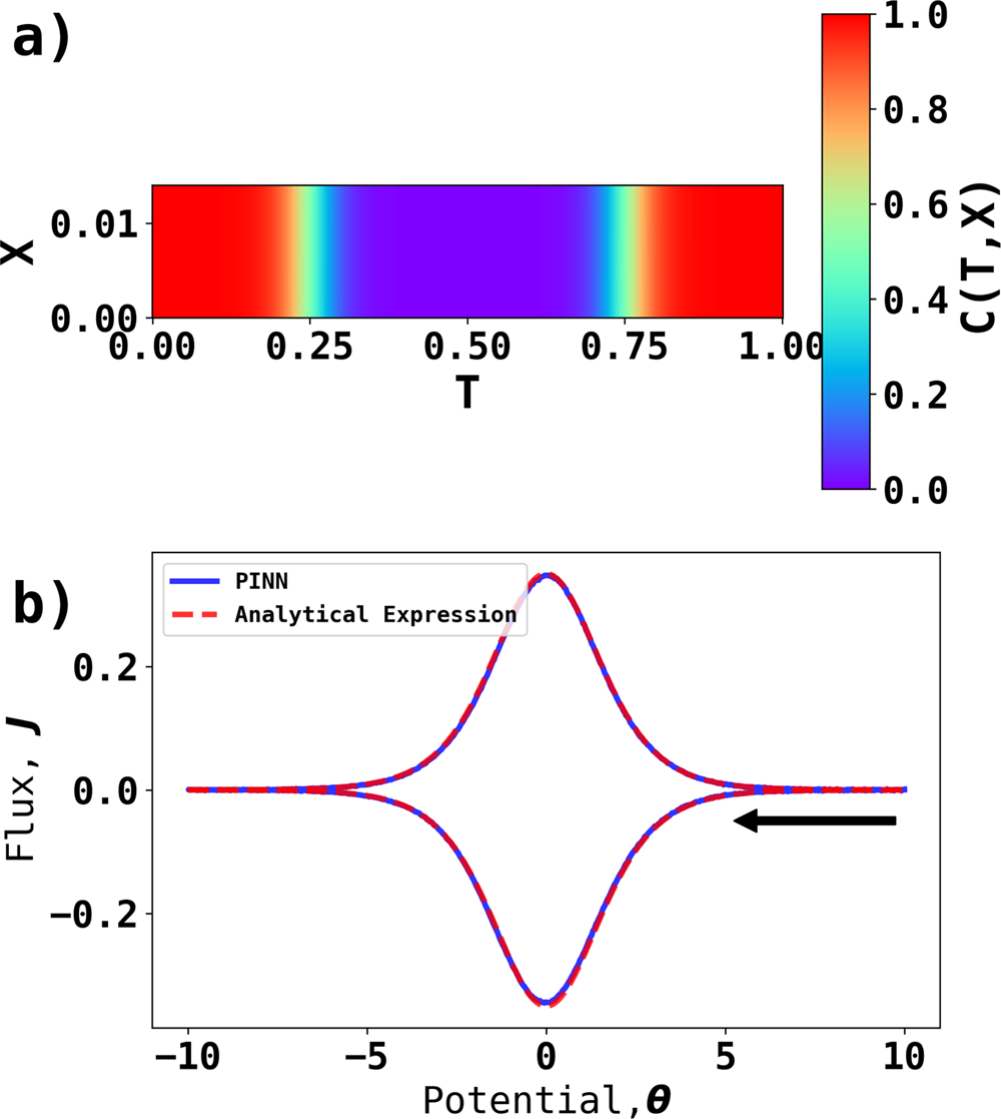
Prediction results of PINN for thin layer linear sweep cyclic voltammetry at σ = 40 and λ = 0.035. (a) Temporal–spatial concentration profile with thin diffusion layer and (b) the voltammogram predicted by PINN (blue line) compared with the voltammogram predicted by eq 13 (red dashed line). The black arrow indicates the start of the initial direction of the scan.

To validate the results of PINN for thin layer cyclic voltammetry, the peak current and voltammogram is compared with analytical expression derived by Hubbard et al.^32^ converted to dimensionless form as
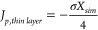
12
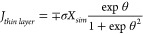
13where *J*_*p*_ is the peak flux for forward scan and *J*_*thin layer*_ describes the shape of voltammogram as a function of dimensionless potential, θ, and the upper and lower signs refer to the cathodic and anodic scans, respectively. As is evident in Figure 3b, the PINN predicted voltammogram closely agrees with the voltammogram predicted by the analytical expression, suggesting that PINN can accurately predict thin-layer voltammetry. To further validate the model, the transition of thin-layer behavior to semi-infinite behavior with increasing λ is illustrated by plotting the dependency on λ of the peak flux, *J*_*p*_, and the peak to peak separation, *θ*_*pp*_. With increasing λ, *J*_*p*_ is expected to transition from the Hubbard equation to the Randles–Ševčík equation and θ_pp_ is expected to increase from θ_*pp*_ = 0 for thin-layer to θ_*pp*_ = 2.218 for semi-infinite behavior.^14^ The plot of *J*_*p*_ vs λ and θ_pp_ vs λ predicted by PINN is shown in Figure 4 and compared with the predictions from simulations using the finite difference method.^14^ The excellent agreement shows that the PINN approach to thin-layer cyclic voltammetry worked well. Figure 4 shows that both PINN and the finite difference method predicts that when λ > 0.8, thin-layer cyclic voltammetry converges to semi-infinite cyclic voltammetry.

**Figure 4:**
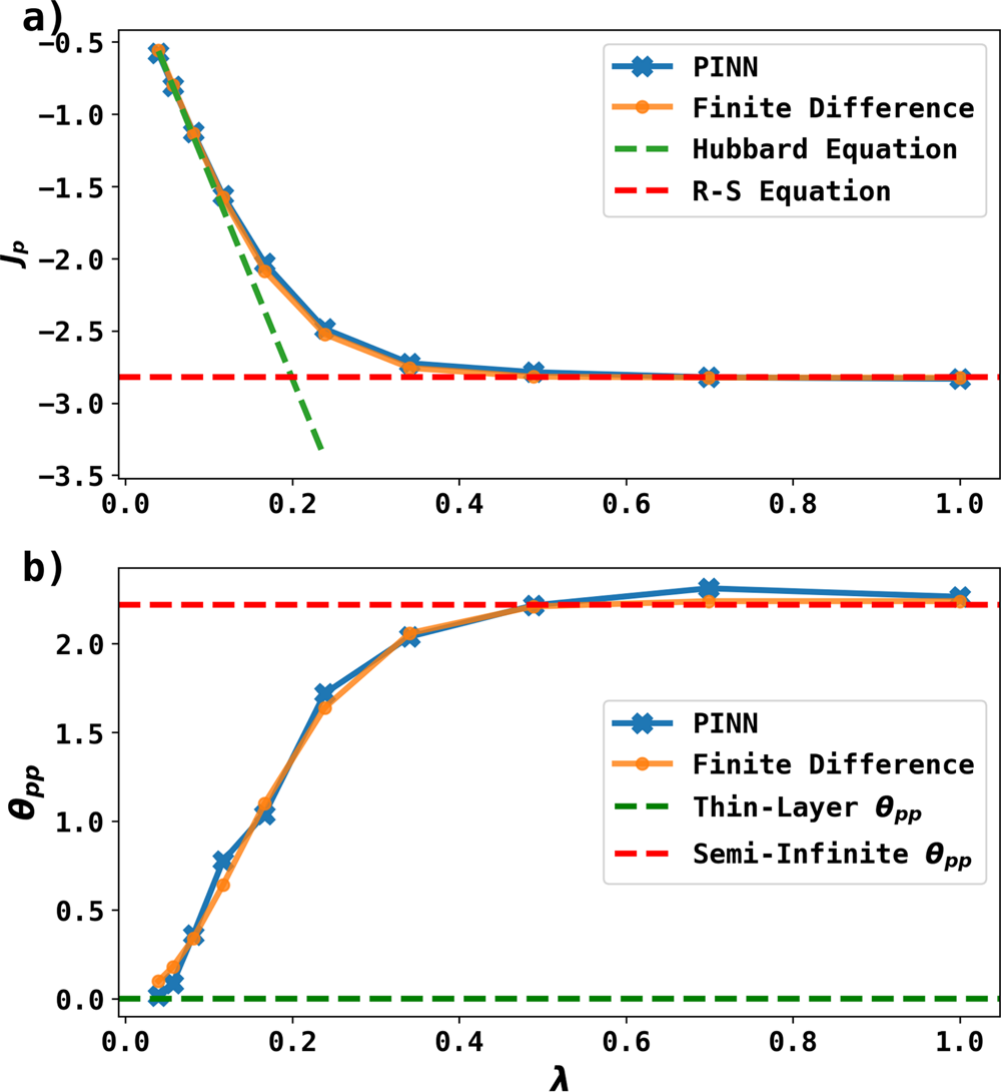
(a) Peak flux predicted by PINN (blue line with marker) and finite difference method (yellow line with marker) as a function of λ for σ = 40. The green and red dashed lines are the currents predicted by the Hubbard equation and the Randles–Ševčík equations, respectively. (b) Peak to peak separation predicted by PINN (blue line with marker) and finite difference method (yellow line with marker) as a function of λ. Green and red dashed lines are expected peak to peak separation for thin-layer and semi-infinite respectively (see text).

To further illustrate and validate the potential applications of PINN in electrochemistry, we explore the PINN prediction for cyclic voltammetry of a microband electrode under semi-infinite diffusion, aiming to predict *C*(*T*,*X*,*Y*) on a temporal-2D spatial domain(. For two-dimensional diffusion eq 8 becomes
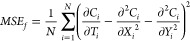
14to account for diffusion in 2D. To enforce eq 14, a set of *N* collocation points {*T*_*i*_,*X*_*i*_,*Y*_*i*_ }_*i*=1_^*N*^ are necessary. A mix of no-flux and fixed concentration boundary conditions are also required to describe the system and can be found in the *Microband Electrode Voltammetry* section in the Supporting Information. To improve optimization and generalization of two-dimensional problems, a learning rate scheduler was utilized to decrease the learning rate by 1% every epoch after 400 epochs.^33^ To reduce the collocation points in the entire domain, the outer boundaries were reduced to  without significant influence to the results. PINN prediction at σ = 4 is performed to reveal the convergent diffusion expected at the microband electrode at lower scan rates. Figure 5 shows a concentration profile when  at the reverse potential *θ*_*switch*_ = −10. Because of the symmetry of the microband electrode, simulating half of the microband is sufficient. The electrode surface is located at *X* = 0,*Y* = [0, 0.5] represented by a flat, red rectangle, and the convergent diffusion is observed at the upper edge of the microband electrode as illustrated in Figure 5a.

**Figure 5:**
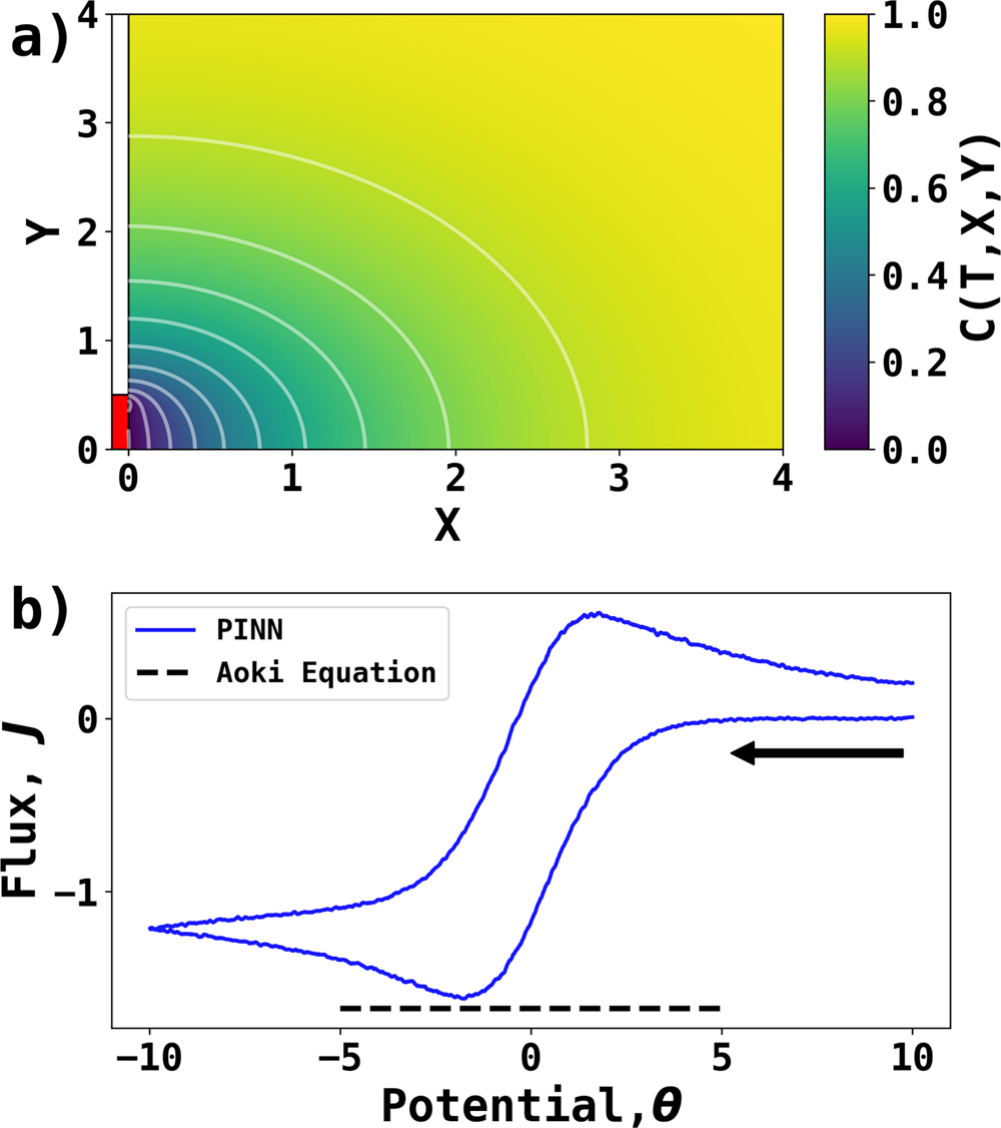
PINN prediction of cyclic voltammetry at a microband electrode for σ = 4. (a) Concentration profile with contours when  and θ = −10 with contour lines of equal concentration. The red block represents the surface of microband electrode. (b) Peak current of cyclic voltammogram (blue) predicted by PINN compared with the peak current predicted by the Aoki equation. The black arrow indicates the start the initial direction of scan.

To validate the prediction by PINN, the peak current of linear sweep voltammetry at microband electrode predicted via FD simulation by Aoki et al.^34^ is
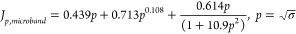
15which is reported accurate to within 2.1% error. The voltammogram can be extracted from the gradients at the electrode surface in the concentration profiles in the temporal domain, then filtered with a Savitzky–Golay filter^35^ to reduce high-frequency noise. Figure 5b compares the PINN predicted voltammogram with the Aoki equation. The peak flux predicted by PINN is 3.3% higher than the Aoki equation, showing the PINN can solve the temporal–spatial problem for 2D simulation of microband electrode within an acceptable level of error. The PINN predictions take approximately 4 h with Nvidia P100 16G GPU.

Last, we employ a PINN to generate new physical insights into the current distributions at nonuniformly accessible electrodes under electrochemically reversible conditions. Specifically, we investigate a model the previously unexplored voltammetric case in which species A and B undergo two-dimensional diffusion in the (*x*, *y*) plane with a square electrode located flat in the plane so that electrolysis is confined to the edges of the electrode. In this way, the consequences to the electrode current distribution caused by the increased diffusional accessibility at the electrode corner were compared to the location on the edges distant from the corner. Interestingly, a clear maximum in the local flux will be seen, but not at the electrode corner as might be expected, and the insights have value for nanoparticle electrochemistry.^36−38^ Specifically, we explore a PINN solution of cyclic voltammetry at the edges of the square electrode. As above, the two-dimensional diffusion equation is enforced as the mode of mass transport for PINN. Two computational subdomains of diffusion equations are configured to obtain better accuracy and faster training.^39^ The square electrode has two electrochemically reaction edges with a Nernstian boundary condition. Details of implementation of other no-flux and fixed concentration boundary conditions and an illustration of the PINN domains can be found in the *Voltammetry at the Edges of a Square Electrode* section in the Supporting Information. The PINN prediction of the concentration profile and cyclic voltammetry at the square electrode with two electrochemically active edges were performed at σ = 40, with the dimensionless edge length set as 1. Figure 6a illustrates the concentration profile at  and θ = −10. Figure 6b shows the flux density at the edges when  as a function of *X* and *Y* coordinates for top and right edges, respectively. The scatter plots showed a small amount of noise possibly due to the stochastic nature of the neural network. The overlapping flux densities on the two edges reflect the symmetry of the square electrode, and thus, they partly validate the predictions. Interestingly, the magnitude of the flux density is largest for an *X*/*Y* coordinate of around 0.8, instead of exactly at the corner of the particle. Figure 6c shows the corresponding voltammograms inferred for the two edges that are almost identical, again confirming the validity of the PINN approach. The occurrence of the maximum current near but not at the corner was unexpected and shows the value of PINN simulations for extracting new semiquantitative physical insights. In the present case the location at the maximum reflects the increased diffusional accessibility near the corner as expected but also highlights, as inferred with hindsight, that the competition between the two edges plays a significant role.

**Figure 6:**
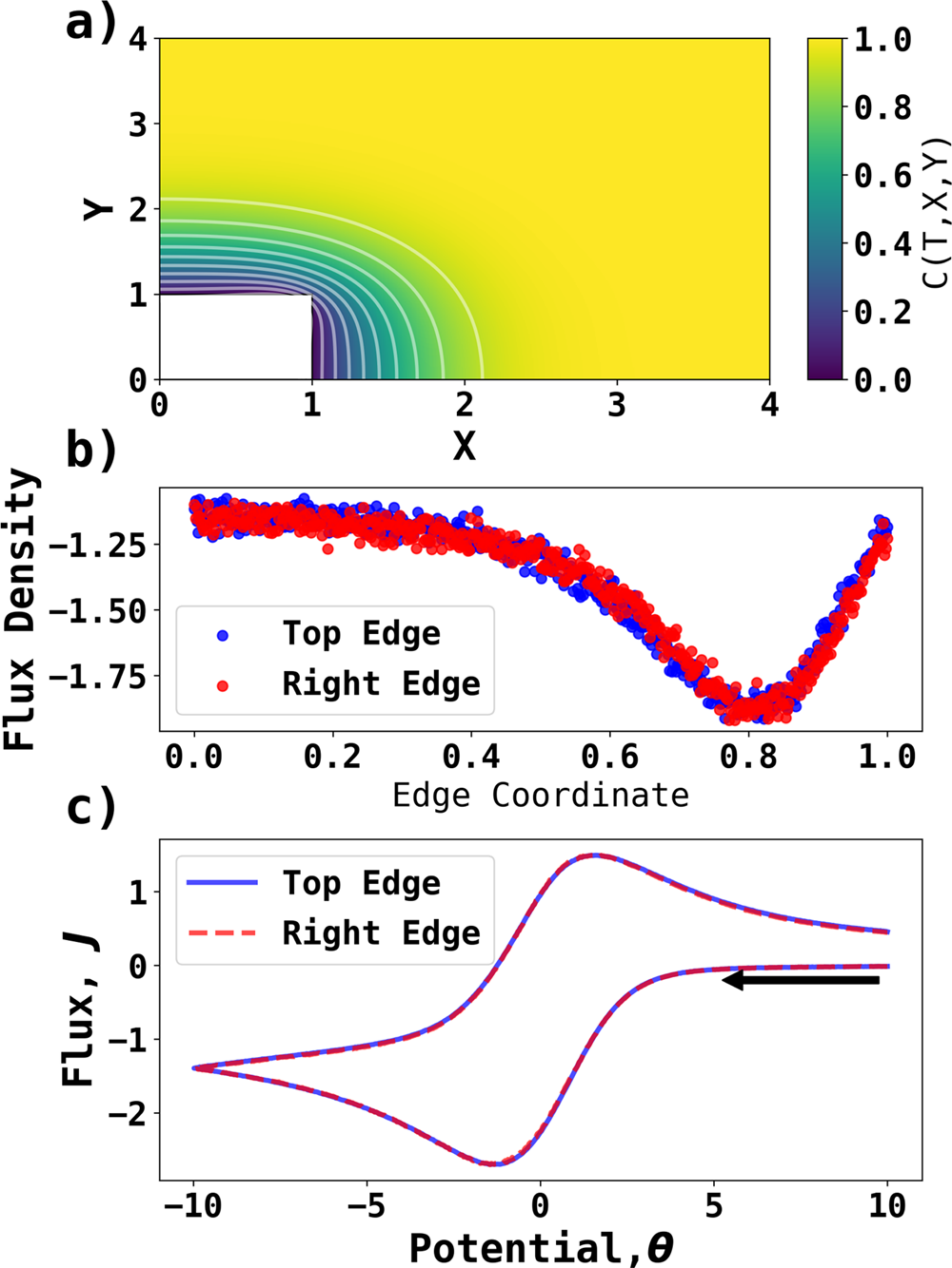
PINN prediction of cyclic voltammetry on the edges of a square electrode at σ = 40. The square electrode has two electrochemically active edges. (a) Concentration profile at  with contour lines of equal concentration. (b) Flux density at the top and right edges when . (c) Cyclic voltammogram of the two edges. The black arrow indicates the start the initial direction of scan.

In summary, in the present work we have developed PINN to solve four problems: two one-dimensional cases including voltammetry with a semi-infinite or a thin-layer boundary condition and two two-dimensional simulations including the microband electrode and the square electrode. The resulting voltammograms are, where available, compared with analytical expressions or voltammograms simulated using finite difference method, proving that PINNs can correctly predict voltammetry, without using any discretization, but with only information on the underlying laws of physics, boundary conditions, and the initial state. When training PINNs to predict voltammograms, we found three major advantage of PINNs. First, with well-established neural network framework like TensorFlow or PyTorch, constructing a neural network is far less daunting than discretizing equations for finite difference or finite element methods. The implication of this is that the investigator can focus on considering the physical models rather than on the mechanics of solution. Instead they simply need to formulate the relevant transport equations and boundary conditions so facilitating a greater range of models to be explored. Second, we found that debugging and modifying PINN programs are relatively easy and relatively rapid. Users can pause the neural network with model check point and generate the concentration profile for visual or mathematical inspection. By generating new data sets and slightly modifying the loss function, PINNs can easily adapt to new prediction tasks. Third, PINNs, as a subclass of neural network, can be easily trained on GPU or CPU with minimal modification and less picky regarding computer hardware and are usually trained on GPU to take advantage of recent advances in GPU computing powers. In addition, PINNs can utilize transfer learning from trained neural network for possibly significant time saving. PINN can be applied to higher dimensions with greater ease, though exponentially more collocation points are necessary to spread the higher dimensional domain. Furthermore, hyperparameters for neural networks, like epochs, learning rate, loss weights, and choice of optimizers can also be tuned for better results. The success of PINN in predicting cyclic voltammetry as shown above signals further possible applications to solution of mass (or heat) transfer problems probably coupled with chemical or electrochemical kinetics, providing a powerful (and likely simpler) alternative to the conventional finite difference or finite element methods.

## Simulation Methods

The PINN program was written in Python using the TensorFlow framework on a workstation with Intel 6800K CPU with 32 GB of RAM and a Nvidia P100 16 GB graphic card. The program is available at https://github.com/nmerovingian/PINN-CV with weights of neural networks for user’s convenience. The validation voltammogram for 1D voltammetry was written in Python and the diffusion equation was solved using finite difference method by discretization of both temporal and spatial domains, and the resulting multidiagonal matrix was solved using the Newton–Raphson method.^14^

## Supporting Information Available

Microband electrode voltammetry and voltammetry at the edges of a square electrode (PDF)
