# Supplementary material for: Predicting Voltammetry Using Physics-Informed Neural Networks
Source: J Phys Chem Lett. 2022 Jan 10;13(2):536–43. doi: 10.1021/acs.jpclett.1c04054 (PMC9084599; doi:10.1021/acs.jpclett.1c04054)
Supplement: Supplementary file 1 — jz1c04054_si_001.pdf [file jz1c04054_si_001.pdf]

Supplementary Information for "Predicting Voltammetry using Physics-Informed Neural Networks"

Haotian Chen[a], Enno Kätelhön[b], Richard G. Compton[a]*

[a] Department of Chemistry, Physical and Theoretical Chemistry Laboratory, Oxford University, South Parks Road, Oxford OX1 3QZ, Great Britain

[b] MHP Management- und IT-Beratung GmbH, Königsallee 49, 71638 Ludwigsburg, Germany

* Corresponding author.

Email address: Richard.compton@chem.ox.ac.uk (R. G. Compton)

Contents

Microband Electrode Voltammetry 1

Voltammetry at the Edges of a Square Electrode...3

Microband Electrode Voltammetry

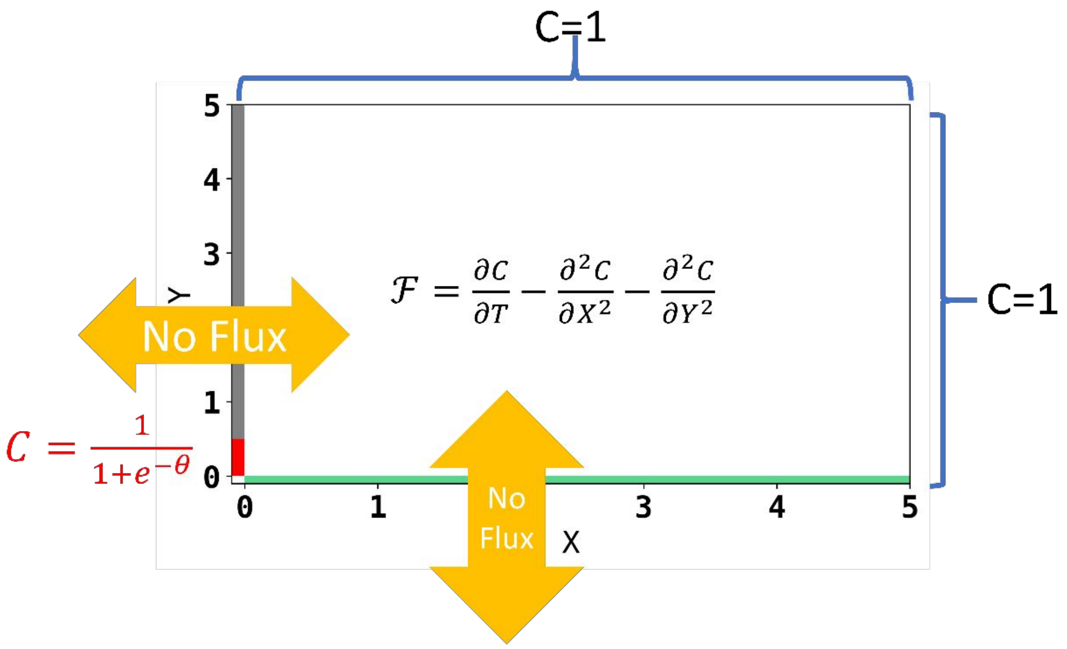

Figure S 1. Illustration of the system for the PINN prediction of cyclic voltammetry at a microband electrode. The small red rectangle represents the surface of electrode and the longer grey rectangle on the Y axis represents an insulating surface. The long green rectangle on the X-axis represents the no-flux boundary condition due to symmetry of the microband electrode. Five boundary conditions are implemented including one boundary condition enforcing the Nernst equation at the electrode surface, two no-flux boundary conditions and two fixed concentration boundary conditions.

The model used for the simulation of cyclic voltammetry at a microband electrode is shown in Figure S 1. The surface of the microband electrode is located at $X = 0, Y = [0,0.5]$ and the concentration at the surface of electrode is determined by the Nernst equation. The no-flux concentration at $X = 0, Y = [0.5, Y_{sim}]$ corresponds to the insulating surface of the electrode. Because of the symmetry of the microband electrode, simulating half of the microband is sufficient and so a no-flux boundary condition is applied at $X = [0, X_{sim}], Y = 0$. Since X_{sim} and Y_{sim} are assumed large enough so the boundaries far from the electrode are unaffected by electrochemical reaction at the surface of electrode, the concentration at $X = [0, X_{sim},], Y = Y_{sim}$, and $X = X_{sim}, Y = [0, Y_{sim}]$ are always 1. In Figure S.1 X_{sim} and X_{sim} take the values of 5.

The PINN prediction of 2D voltammetry at microband electrode requires solution of a temporal-spatial differential equation by predicting $C(T,X,Y)$ of the whole simulation domain. Illustration of simulation scheme is shown within Figure S 1. The diffusion equation and boundary conditions are:

$$\frac{\partial C}{\partial T} - \frac{\partial^2 C}{\partial X^2} - \frac{\partial^2 C}{\partial Y^2} = 0 \qquad \mathcal{T} \times \Omega_X \times \Omega_Y \qquad (1.1)$$

$$C = \frac{1}{1 + e^{-\theta}} \qquad \mathcal{T}, X = 0, Y = [0,0.5] \qquad (1.2)$$

$$\frac{\partial C}{\partial X} = 0 \qquad \mathcal{T}, X = 0, Y = [0.5, Y_{sim}] \qquad (1.3)$$

$$C = 1 \qquad \mathcal{T} \times \Omega_X, Y = Y_{sim} \qquad (1.4)$$

$$C = 1 \qquad \mathcal{T}, X = X_{sim}, \Omega_Y \qquad (1.5)$$

$$\frac{\partial C}{\partial Y} = 0 \qquad \mathcal{T} \times \Omega_X, Y = 0 \qquad (1.6)$$

$$C = 1 \qquad T = 0, \Omega_X \times \Omega_Y \qquad (1.7)$$

where $\mathcal{T} \in [0, T_{sim}]$, $\Omega_X \in [0, X_{sim}]$, $\Omega_Y \in [0, Y_{sim}]$, representing the temporal and two-dimensional spatial domain respectively. Note that because of symmetry of the microband electrode, only half of the microband electrode is necessary for simulation. The electrode is thus located at $X = 0, Y = [0,0.5]$. Seven training datasets are thus required to enforce the physics law and boundary conditions. For example, to enforce Equation 1.1, a set of N collocation points $\{T_i, X_i, Y_i\}_{i=1}^{N}$ randomly uniformly distributed in the $\mathcal{T} \times \Omega_X \times \Omega_Y$ domain. In neural networks, the diffusion equation, boundary condition and physics law are enforced by a linear combination of mean squared error functions (MSEs) as:

$$\mathcal{L} = w_1 MSE_f + w_2 MSE_{Eqn\_1.2} + w_3 MSE_{Eqn\_1.3} + w_4 MSE_{Eqn\_1.4} + w_5 MSE_{Eqn\_1.5} + w_6 MSE_{Eqn\_1.6} + w_7 MSE_{Eqn1.7} \quad (2)$$

where w_j are hyperparameters for training to balance weights for each MSEs since each MSEs may have different numerical scales. The first MSE_f represents the error of enforcing the two-dimensional diffusion equation as:

$$MSE_f = \frac{1}{N}\sum_{i=1}^{N}\left(\frac{\partial C_i}{\partial T_i} - \frac{\partial^2 C_i}{\partial X_i^2} - \frac{\partial^2 C_i}{\partial Y_i^2}\right)^2 \quad (3)$$

Similarly, to enforce a no flux boundary condition like Equation 1.3 and 1.6, the error functions are configured to enforce no flux in Y direction on the boundary:

$$MSE_{Eqn\_1.3} = \frac{1}{N}\sum_{i=1}^{N}\left(\frac{\partial C_i}{\partial X_i}\right) \quad (4.1)$$

$$MSE_{Eqn\_1.6} = \frac{1}{N}\sum_{i=1}^{N}\left(\frac{\partial C_i}{\partial Y_i}\right) \quad (4.2)$$

For fixed concentration boundary conditions like Equation 1.2, 1.4, 1.5 and 1.7, the error functions are:

$$MSE_{Eqn\_1.2} = \frac{1}{N}\sum_{i=1}^{N}\left(C_i - \frac{1}{1+e^{-\theta}}\right) \quad (5.1)$$

$$MSE_{Eqn\_1.4} = \frac{1}{N}\sum_{i=1}^{N}(C_i - 1) \quad (5.2)$$

$$MSE_{Eqn\_1.5} = \frac{1}{N}\sum_{i=1}^{N}(C_i - 1) \quad (5.3)$$

$$MSE_{Eqn\_1.7} = \frac{1}{N}\sum_{i=1}^{N}(C_i - 1) \quad (5.4)$$

During training, the optimizer minimizes the loss function by tuning the weights and biases of neural network. In the present work, the neural network was trained for 1600 epochs with a learning rate scheduler: the learning rate was decreased by 1% every epoch after 400 epochs.

Voltammetry at the Edges of a Square Electrode

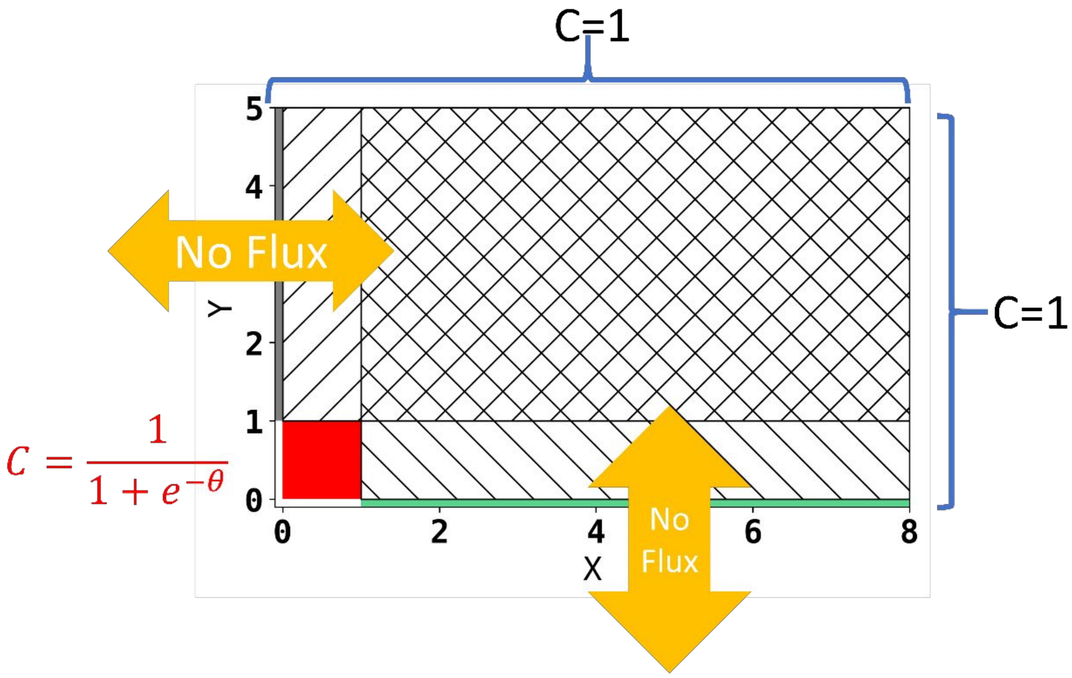

Figure S 2. Illustration of the system for the PINN prediction of cyclic voltammetry at the edges of a square electrode. The red square represents the electrode which has two electroactive edges on the top and right of the square. The longer grey rectangle on the Y-axis represents the no-flux boundary condition on the insulating surface and the green rectangle on the X-

axis represents the no-flux boundary condition because of symmetry. The different hatch styles refer to the different diffusion subdomains used for PINN prediction (see main text)

The approach to the PINN prediction of cyclic voltammetry at the edges of a square electrode is illustrated in Figure S 2.The red square represents the square electrode with two electroactive edges: the top and right edges with Nernstian boundary condition. Similar to the microband electrode, simulation of the square electrode also involves two no-flux boundary condition and two fixed concentration boundary condition.

The PINN prediction uses two subdomains to enforce the diffusion equation as illustrated in Figure S 2; the two subdomains overlap at $X = [1,X_{Sim}] \times Y = [1,Y_{sim}]$. The diffusion equations and boundary conditions are:

$$\frac{\partial C}{\partial T} - \frac{\partial^2 C}{\partial X^2} - \frac{\partial^2 C}{\partial Y^2} = 0 \quad \mathcal{T} \times \Omega_X \times Y = [1,X_{sim}] \quad (6.1)$$

$$\frac{\partial C}{\partial T} - \frac{\partial^2 C}{\partial X^2} - \frac{\partial^2 C}{\partial Y^2} = 0 \quad \mathcal{T} \times X = [1,X_{sim}] \times \Omega_Y \quad (6.2)$$

$$C = \frac{1}{1 + e^{-\theta}} \quad \mathcal{T} \times X = [0,1], Y = 1 \quad (6.3)$$

$$C = \frac{1}{1 + e^{-\theta}} \quad \mathcal{T}, X = 1, Y = [0,1] \quad (6.4)$$

$$\frac{\partial C}{\partial X} = 0 \quad \mathcal{T}, X = 0, Y = [1,Y_{sim}] \quad (6.5)$$

$$C = 1 \quad \mathcal{T} \times \Omega_X, Y = Y_{sim} \quad (6.6)$$

$$C = 1 \quad \mathcal{T}, X = X_{sim}, \omega_Y \quad (6.7)$$

$$\frac{\partial C}{\partial Y} = 0 \quad \mathcal{T} \times X = [1,X_{sim}], Y = 0 \quad (6.8)$$

$$C = 1 \quad T = 0, \Omega_X \times \Omega_Y \quad (6.9)$$

Similar to cyclic voltammetry at a microelectrode, the loss functions are linearly combined to enforce the initial and boundary conditions:

$$\mathcal{L} = \sum_{i=1}^{n=9} w_i MSE_i (7)$$

The mean squared functions for the two subdomains are:

$$MSE_{Eqn\_6.1} = \frac{1}{N} \sum_{i=1}^{N} \left(\frac{\partial C_i}{\partial T_i} - \frac{\partial^2 C_i}{\partial X_i^2} - \frac{\partial^2 C_i}{\partial Y_i^2} \right)^2 \quad (7.1)$$

$$MSE_{Eqn\_6,2} = \frac{1}{N} \sum_{i=1}^{N} \left(\frac{\partial C_i}{\partial T_i} - \frac{\partial^2 C_i}{\partial X_i^2} - \frac{\partial^2 C_i}{\partial Y_i^2} \right)^2 \quad (7.2)$$

The boundary conditions at the surface of electrode is enforced by:

$$MSE_{Eqn\_6.3} = \frac{1}{N} \sum_{i=1}^{N} \left(C_i - \frac{1}{1 + e^{-\theta}} \right) \quad (8.1)$$

$$MSE_{Eqn\_6,4} = \frac{1}{N} \sum_{i=1}^{N} \left(C_i - \frac{1}{1 + e^{-\theta}} \right) \quad (8.2)$$

The other boundary conditions and the initial state are enforced by:

$$MSE_{Eqn\_6,5} = \frac{1}{N}\sum_{i=1}^{N}\left(\frac{\partial C_i}{\partial X_i}\right) \quad (9.1)$$

$$MSE_{Eqn\_6.6} = \frac{1}{N}\sum_{i=1}^{N}(C_i - 1) \quad (9.2)$$

$$MSE_{Eqn\_6,7} = \frac{1}{N}\sum_{i=1}^{N}(C_i - 1) \quad (9.3)$$

$$MSE_{Eqn\_6.8} = \frac{1}{N}\sum_{i=1}^{N}\left(\frac{\partial C_i}{\partial X_i}\right) \quad (9.4)$$

$$MSE_{Eqn\_6.9} = \frac{1}{N}\sum_{i=1}^{N}(C_i - 1) \quad (9.5)$$

The neural network was trained for 400 epochs.
